# Enlarged Glands

**Published:** 1890-04-12

**Authors:** 


### ENLARGED GLANDS.

Mr. Edmund Owen in his Lettsomian lecture, recently delivered at the Medical Society of London, selected certain subjects in the surgery of infancy and childhood as his theme. Enlarged glands, especially those of the neck, were accorded considerable attention. As regards iodine, Mr. Owen says the chief virtue resides in the stain which it makes. After a prolonged course of such application the gland begins to grow soft, and the second part of routine treatment is entered upon, viz., a long series of poulticing. But probably, if the medical attendant advises a more radical treatment, it is declined, either from dread of operation or fear of scarring. But we should no more leave these cases of enlarged glands to Nature, than a curious tooth or a suppurating joint. Mr. Owen does not, however, ignore the influence change of air and good sanitary conditions may have on enlarged glands, and of all places he commends Bournemouth, especially in cases where no cause for the enlargement can be discovered. But if a gland goes on increasing in size, while the glands in the chain next above it and below it begin to swell, the sooner that all three are weeded out the better. The hair should be cut short, and the side of the neck thoroughly cleansed. Chloroform having been administered, a sufficiently free incision is made over the tumour, most likely along the anterior border of the sterno-mastoid. The gland is then exposed by working through the intermediate stratum with a steel director and forceps. Vessels entering the gland are torn through with two forceps. Mr. Owen considers this method better than dissecting the glands out. After removal of the glands the cavity is syringed with weak mercuric solution, and horsehair sutures applied, previously asepticised. A scrap of moist protective is placed over the wound, and then a pad of wood-wool. No pillow is allowed in the cot, but a small junk is placed beneath the occiput, and the head is steadied by sand-bags. From this position the child is not allowed to stir, either for feeding or for any other purpose. The dressings are removed next day, also most of the sutures, by which procedure suture marks are avoided. When the child is allowed to get up his neck should be still kept at rest with a stiff stock. If the operation has been long delayed and pus escapes, the best that can be done is to lay open, scrape out, and irrigate the cavity. In the Retrospect, November 2nd last, under the head Scrofula, Mr. Treeves' operation for enlarged glands was noticed. Mr. Treeves prefers rather to cut out the glands, than working with steel director and forceps.

